# Book Review

**DOI:** 10.1120/jacmp.v6i4.2182

**Published:** 2005-11-22

**Authors:** Christopher H. Cagnon

**Affiliations:** ^1^ Assistant Professor of Radiological Sciences David Geffen School of Medicine at UCLA

## Abstract

*Radiology Review: Radiology Physics* by Ed Nickoloff, Copyright 2005, Elsevier & Saunders, ISBIN 1‐4160‐2260‐0, List Price $59.95 (paper back)

For better or for worse, students preparing for impending board exams typically seek some form of condensed review material, and they absolutely clamor for sample test questions. A reasonably comprehensive review text presents the allure of a one‐stop‐shopping study guide, and a variety of sample questions allows the examinee to gauge their comprehension of the subject material, and perhaps if they're very lucky, actual exam questions will be similar, if not identical, to sample questions.

Of course, the advantages of the condensed review are mutually exclusive to its shortcomings. The more consolidated the review (and therefore efficient as a study guide), the less comprehensive coverage of the material it can offer. Similarly, being able to answer questions recalled from prior exams or offered in a review text does not necessarily guarantee success in future exams. By definition, a review is renewed study of previously studied material and is not intended as the primary or sole source of subject content on a study syllabus. The danger of course is that it often is; particularly in Radiology residency training programs where learning the principals of imaging physics competes with a myriad other clinical, anatomical, and pathological lessons.

*Radiology Review: Radiologic Physics* is a well written, easy reading, reference guide that ‐ as advertised ‐ reviews the subjects of X‐ray production, x‐ray interactions with matter, image quality, quality control, radiation safety and dosimetry, and regulatory requirements. It covers imaging modalities including radiography, mammography, fluoroscopy, computed tomography, magnetic resonance imaging, ultrasound, and nuclear medicine. Each topic is presented in a chapter with approximately five pages of consolidated subject material followed by 20–30 relevant questions and answers. It is thus similar in content and format to Huda and Slone's *Review of Radiologic Physics* and both are targeted towards Radiology residents preparing for board examinations. What it offers in addition is a “bonus CD‐ROM” with 46 PowerPoint presentations comprising 1500 slides. These are billed as supplements of the written material to clarify difficult concepts. In practice, without the accompanying narrative, these slides are likely to be of more use to an instructor preparing courses than to the student.

A difficulty for any text of Radiology imaging is keeping up with evolving technology. As advertised, *Radiology Review: Radiologic Physics* includes information on computed and digital radiography, computers, PACS, and PET as do newer editions of Huda's review. However, a student interested in the advances and clinical implications of multi‐detector CT, MR spectroscopy and functional imaging, or PET/CT should look elsewhere.

Lastly, the preface of *Radiology Review* also recommends the book for graduate students and suggests that, with the CD, it might be used as an individual study guide in lieu of a formal Radiology physics course. In this I disagree. A Medical Physics graduate student should view use this text as its title suggests: a consolidated review and a source of practice questions. Precisely because of the nature of its condensed material and simplification of concepts it should not be used as a substitute for comprehensive, conceptual understanding of its subject content. *Radiology Review* will certainly and justifiably be popular with residents, technologists, and even graduate students preparing for board exams. It just should not serve as the solitary source of subject knowledge; not for the Radiology resident and certainly not for the graduate student.

